# Anisotropic Ion Conducting Particulate Composites for Bioelectronics

**DOI:** 10.1002/advs.202104404

**Published:** 2022-01-27

**Authors:** Dickson R. Yao, Han Yu, Onni J. Rauhala, Claudia Cea, Zifang Zhao, Jennifer N. Gelinas, Dion Khodagholy

**Affiliations:** ^1^ Department of Electrical Engineering Columbia University New York NY 10027 USA; ^2^ Department of Neurology Columbia University Medical Center New York NY 10032 USA; ^3^ Institute for Genomic Medicine Columbia University Medical Center New York NY 10032 USA

**Keywords:** anisotropic electrolyte, integrated organic electrochemical transistors, organic bioelectronics

## Abstract

Acquisition, processing, and manipulation of biological signals require transistor circuits capable of ion to electron conversion. However, use of this class of transistors in integrated sensors or circuits is limited due to difficulty in patterning biocompatible electrolytes for independent operation of transistors. It is hypothesized that it would be possible to eliminate the need for electrolyte patterning by enabling directional ion conduction as a property of the material serving as electrolyte. Here, the anisotropic ion conductor (AIC) is developed as a soft, biocompatible composite material comprised of ion‐conducting particles and an insulating polymer. AIC displays strongly anisotropic ion conduction with vertical conduction comparable to isotropic electrolytes over extended time periods. AIC allows effective hydration of conducting polymers to establish volumetric capacitance, which is critical for the operation of electrochemical transistors. AIC enables dense patterning of transistors with minimal leakage using simple solution‐based deposition techniques. Lastly, AIC can be utilized as a dry, anisotropic interface with human skin that is capable of non‐invasive acquisition of individual motor action potentials. The properties of AIC position it to enable implementation of a wide range of large‐scale organic bioelectronics and enhance their translation to human health applications.

## Graphical abstract

It is shown that it is possible to eliminate the need for electrolyte patterning by enabling directional ion conduction as a property of the material serving as electrolyte. The anisotropic ion conductor (AIC) is a soft, biocompatible composite material comprised of ion‐conducting particles and an insulating polymer. AIC allows dense patterning of transistors and sensors with minimal leakage and cross‐talk.

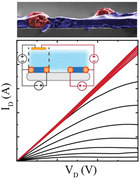

## 1 Introduction

Movement of ions is critical for generation of biopotentials from electrically active cells in tissue. Soft, biocompatible electronic components that directly interact with ions offer opportunities for effective, biologically‐based sensing and processing. Transistors serve as the backbone for most circuits, and various architectures have been designed to interface with tissue and create active bioelectronics.^[^ ^1^, ^2^, ^3^ ^]^ Of these, transistors that are made from organic semiconductors and conducting polymers, such as electrolyte‐gated organic field effect transistors (EGOFET), organic electrochemical transistors (OECT), and internal ion‐gated organic electrochemical transistors (IGT), are modulated by ionic fluctuations in electrolyte that arise via electric double‐layer capacitance and redox reactions.^[^ ^4^, ^5^, ^6^, ^7^, ^8^, ^9^, ^10^, ^11^ ^]^ However, the ability to use these transistors as integrated sensors or circuits is limited due to difficulty establishing independent gating of individual transistors. Multiple approaches have been employed to address this issue, but substantial challenges remain. For instance, high performance EGOFETs have been used to form integrated circuits by photopatterning or printing ionic gels over each transistor.^[^ ^12^, ^13^, ^14^ ^]^ This method is complex to implement and substantially lowers the maximum transistor density due to poor spatial patterning resolution of the ionic gel. OECT‐based integrated circuits can be generated using screen printing, but devices cannot be miniaturized, and the centimeter‐scale circuits have temporal response in the regime of seconds, which precludes use in most temporally sensitive applications.^[^ ^15^, ^16^ ^]^ IGTs can create fast, miniaturized circuits by incorporating ions within the channel bulk and using an ion membrane to allow an ionic path between gate and channel.^[^ ^9^, ^10^ ^]^ The ion membrane needs to be patterned, introducing complexity into the fabrication process and limiting density. To permit electrolyte patterning with any of these approaches, crosslinkers in the form of photo‐initiators are often used, yet these additives also lower ion mobility and therefore reduce transistor performance.^[^ ^17^ ^]^ A similar obstacle is encountered when attempting high spatial resolution biopotential sensing from tissue, as the conductive interfacing gels cannot be patterned to allow independent signal acquisition from closely spaced sensors.^[^ ^18^, ^19^ ^]^ Mixed conducting particulate composites (MCPs) can substantially increase the spatial resolution for such sensing. However, they form a wet interface and rely upon conformability of the bioelectronic device for reliable mechanical contact.^[^ ^20^ ^]^

We hypothesized that it would be possible to eliminate the need for electrolyte patterning and a wet sensing interface by incorporating directional ion conduction as a property of the material serving as electrolyte. Here, we introduce a soft, biocompatible composite material comprised of ion‐conducting particles and an insulating scaffolding polymer, the anisotropic ion conductor (AIC). A wide range of materials are amenable to creation of AICs, and the physical processes used are scalable, solvent‐free, and tunable for various applications. AIC integrates well with organic transistors because it permits swelling of the polymeric channel for high performance operation. We generated effective AIC‐based integrated circuits with minimal leakage at high transistor density using simple solution‐based deposition techniques without necessity for any patterning. AIC also formed a dry, anisotropic interface with human skin that was mechanically stable and enabled non‐invasive acquisition of high spatiotemporal resolution electromyography (EMG) signals at the level of individual motor action potentials. Therefore, AIC offers a simple, pattern‐free solution for implementation of biocompatible, miniaturized, high density integrated organic circuits.

## 2 Results

**Figure 1:**
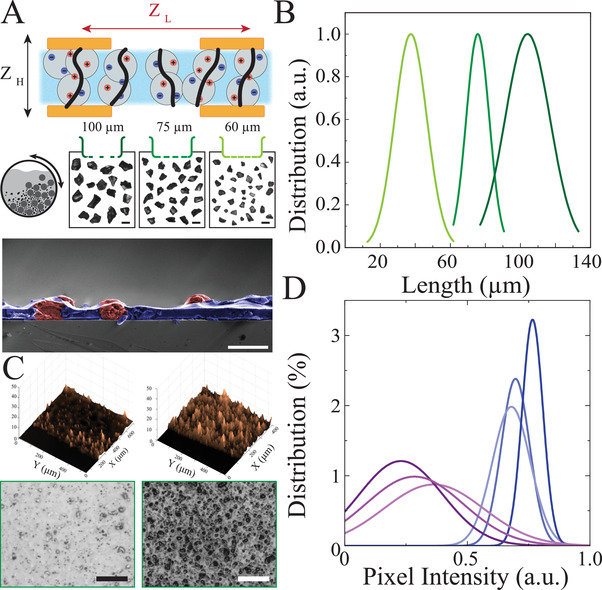
Structure and synthesis of ion conducting particulate composites with controllable particle size. A) Top: Schematic illustration of AIC cross‐section composed of ions (red and blue), conducting particles (gray), and insulating scaffold (blue); black lines indicate the vertical conduction path (top). Middle: Schematic of stainless steel ball mill grinding a mixture of ion‐conducting particles suspended in isopropyl alcohol. Dried particles are filtered through sieves with various mesh sizes to obtain the range of desired particle sizes. Micrographs show various NaPSS particle sizes after dry sieving. Scale bars 100 µm (middle). Bottom: Scanning electron microscopy (SEM) cross‐sectional image of NaPSS/PU film (particle size, *H* = 10 µm, 20% w/v concentration). False colors highlight the NaPSS particles (red) and the PU scaffold (purple). Scale bar 10 µm (bottom). B) Particle size distributions of NaPSS particles filtered through 100, 75, and 60 µm mesh openings (dark to light colors), obtained by optical microscopy. C) 3D mechanical profilometry measurement of the thickness and roughness of NaPSS/PU AIC films (*H* = 10 µm) at 10% and 60% w/v concentrations (top). Corresponding optical microscope images of NaPSS/PU composites (*H* = 10 µm) at 5% (left) and 40% (right) w/v concentrations (bottom). Scale bars 100 µm. D) Pixel intensity distributions of NaPSS/PU films (particle size = 10 µm) at 5% (replicates in shades of purple) and 40% (replicates in shades of blue) w/v concentrations demonstrating that particles are uniformly spread throughout different regions of the composite films. Pixel intensity is analyzed from images captured using an optical microscope at 50× magnification.

We hypothesized that we could create an anisotropic ion conductor with tunable ion conduction and insulation material properties, rather than relying on a patterning process during fabrication to achieve this functionality. Such a material would support ionic conduction across its thickness, while serving as an ionic barrier laterally. To test this hypothesis, we aimed to synthesize 2‐phase particulate composites that contained ion conducting particles embedded in an insulating scaffold (**Figure** **1**a; top). Controlling the size and density of the ion‐conducting particles within the scaffolding polymer would thereby allow tuning of the ion conducting properties of the material. We created ion conducting particles by wet ball milling various polymers, such as sodium polystyrene sulfonate (NaPSS). We then sieved the particles with controllable upper and lower boundaries to achieve a defined range of particle sizes (*H*, Figure 1a; middle). The particle size distribution was measured using optical imaging and adaptive thresholding to localize the particles’ area and axis. We found that the sieving process led to selective narrow distributions of particle sizes across a range of 1–150 µm (Figure 1b, Figure S1a and Table S1, Supporting Information). We mixed the particles with insulating polymers, such as polyurethane (PU), to establish particulate composite films (Figure 1a; bottom, Figure S1b, Supporting Information). We utilized 3D mechanical profilometry to establish a topography image of the film over a large area, demonstrating film roughness and thickness comparable to individual particles and highlighting non‐coagulated particles inside the insulating layer (Figure 1c). It is worth noting that smaller particle sizes can lead to a lower surface roughness (Figure S2, Supporting Information). Next, we examined the uniformity of particle placement, as this has ramifications for the applicability of the material for large‐scale electronics and biosensors. Optical microscopy combined with image processing techniques to differentiate particles from the insulating layer was used to determine the distribution profile of particles in large‐scale films (Experimental Section). We obtained multiple images at different locations from the samples, and computed histograms of the gray‐scale pixels (Figure 1d, Figure S3 and Table S2, Supporting Information). The unimodal histograms indicated the uniform distribution of particles within the scaffold at any given concentration. Therefore, this physical processing and synthesis of particles allow scalable production of 2–phase, particulate composite films with controllable ion‐conducting particle size and density.

**Figure 2:**
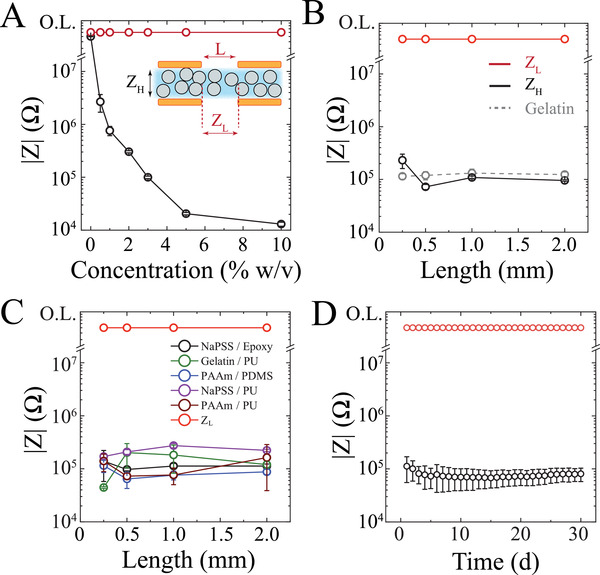
Ion conducting particulate composites can be created from a variety of materials and result in highly anisotropic ion conducting (AIC) films. A) Electrochemical impedance spectroscopy (EIS) measurements (1 Hz) of NaPSS/PDMS AIC films (*H* = 100 µm) demonstrate tunable out‐of‐plane impedance (*Z*_H_) as particle concentration varies, while in‐plane impedance (*Z*_L_) remains high. In‐plane impedances are measured between Au electrodes spaced *L* = 250 µm apart. O.L., overload. Inset: cross‐sectional schematic illustrating an AIC layer (blue and gray) sandwiched between 4 Au‐based electrodes (orange). *Z*_H_ and *Z*_L_ denote vertical and lateral impedances, respectively. B) Contrast between the impedances of an isotropic ion conductor (gray, dashed line) of 10% w/v gelatin in deionized water and NaPSS/PDMS AIC (solid line; *H* = 100 µm, 10% w/v). The vertical impedance (black) of the isotropic conductor is comparable to the vertical impedance of AIC. However, the gelatin‐based isotropic conductor has horizontal impedance comparable to its vertical impedance, while AIC is selectively vertically conducting due to insulating material separating the ion conducting particles. O.L., overload. C) AIC films are achievable with a wide variety of materials. Ion conducting particles included NaPSS, gelatin, and polyacrylamide (PAAm). Insulating polymer scaffolds included polyurethane (PU), polydimethylsiloxane (PDMS), and epoxy. In‐plane impedance (red) is significantly higher than the through‐plane impedance of various AIC materials: NaPSS/epoxy (black), gelatin/PU (green), PAAm/PDMS (blue), and NaPSS/PU (purple), PAAm/PU (brown). O.L., overload. D) Anisotropic ion conductivity persisted for at least 30 days as demonstrated by vertical impedance (black) remaining several orders of magnitude lower than lateral impedance (red) over this period. PAAm/PU AIC films (*H* = 20 µm, 10% w/v concentration) are encapsulated such that hydration is entrapped, and an aqueous environment is maintained. O.L., overload.

To evaluate and characterize the anisotropic properties of these films, we established an experimental setup where the particulate film was sandwiched between two glass substrates each patterned with Au‐based contact pads (**Figure** **2**a; inset). This setup enabled concurrent measurement of lateral (*Z*_L_) and vertical impedances (*Z*_H_) to directly assay ion conduction from the sample (Figure S4, Supporting Information). We then compared these two impedance values across various particle concentrations for a given particle size and found that higher particle concentrations lowered the impedance by several orders of magnitude (Figure 2a, Figure S5a, Supporting Information). Further increasing particle concentration was limited by difficulties in obtaining processible films of uniform thickness and did not result in a substantial additional impedance decrease. Film thicknesses were kept in proportion to particle size, though thicker films up to 2 times the particle size produced similar impedance results (Figure S5b,c, Supporting Information). We next examined lateral distances across which the films maintain anisotropy. We microfabricated Au‐based pads on glass substrates with various spacing (250–2000 µm), deposited the AIC film on one substrate, and laminated the second substrate over the AIC film with aligned contacts. The AIC maintained anisotropy (|*Z*_L_| / | *Z*_H_|) higher than 10^3^ at lateral distances approximately 2 times its particle size at 10% w/v concentration (Figure 2a,b). The high vertical ionic conductivity of AIC is similar to a homogenous hydrogel‐based ion conductor such as gelatin (Figure 2b; dashed gray line). Moreover, the physical processes used to prepare the AIC are scalable and can be applied to variety of 2‐phase particulate composites formations including: NaPSS, poly(acrylamide) (PAAm) and gelatin as ion conducting particles with polydimethylsiloxane (PDMS), polyurethane (PU) and epoxy groups as ion barriers (Figure 2c). Lastly, we performed impedance measurements longitudinally and established that AIC is highly stable and maintains its anisotropic properties over at least 30 days (Figure 2d). The combination of PAAm and PU was chosen to provide the best hydration/swelling stability for long‐term applications. As a scaffold, PU is a thermosetting polymer with low swelling in water compared to PDMS, a soft elastomer. Additionally, PAAm particles are crosslinked hydrogels that remain physically embedded into the scaffold.

**Figure 3:**
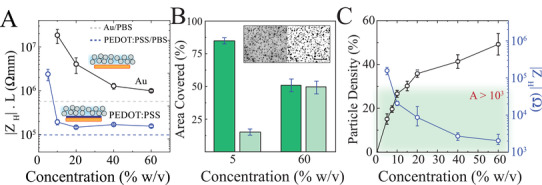
AIC allows effective hydration of conducting polymers. A) Through‐plane impedance (1 Hz) normalized by electrode length of NaPSS/PU AIC (*H* = 10 µm) at various particle concentrations, measured via EIS on gold electrodes (black) and on PEDOT:PSS‐coated electrodes (blue, solid). For reference, the impedance of 10% w/v gelatin in deionized water (gray, dashed) and 1× PBS (blue, dashed) measured on PEDOT:PSS‐coated electrodes are shown. Symbols represent mean ± standard error. B) Breakdown of the percentage of surface area covered by NaPSS particles (light green) and by the PU scaffold material (dark green) of AIC (*H* = 10 µm) at two contrasting particle concentrations (bars denote ± standard error). Inset: optical micrograph of the corresponding 5% w/v AIC film (left) and the binarized image (right) highlighting NaPSS particles (black) and the PU scaffold (white). Scale bar 100 µm. C) Particle density (black curve) and its resultant vertical electrochemical impedance (1 Hz; blue curve) as a function of particle concentrations (symbol notes mean ± standard error). Green shaded area highlights where anisotropy (*A* = |*Z*_L_| /|*Z*_H_|) is above 10^3^.

In order for AIC to become an effective anisotropic medium for bioelectronics and ion‐driven devices, it should in addition to providing ion conduction allow efficient water transport. This property is especially important for electrochemical and electrolyte‐gated devices, where the channel current modulation relies on the formation of electric double layer (EDL) capacitance and redox reactions.^[^ ^7^, ^21^, ^22^, ^23^ ^]^ Additionally, water‐based swelling of conducting polymers is critical to establish volumetric capacitance for organic electrochemical transistors (OECTs).^[^ ^24^, ^25^, ^26^, ^27^ ^]^ To evaluate the ability of AIC to transport and allow water uptake by the underlying polymeric particles, we prepared AIC with various particle concentrations and deposited films onto Au‐based and conducting polymer‐based (poly(3,4‐ethylenedioxythiophene), PEDOT:PSS) electrodes to perform electrochemical impedance spectroscopy (**Figure** **3**a; inset). The AIC coated Au‐based electrode exhibited an inverse relationship between impedance and particle concentration (Figure 3a; black trace). Because Au is a hard, non‐swelling electrode material, the increase of particle density enlarged the effective surface area, and lowered impedance (|Z| ≈ 1 / area). In contrast, the conducting polymer‐based electrode showed a constant and lower impedance value, comparable to isotropic electrolytes,^[^ ^28^, ^29^ ^]^ for a large span of particle concentrations (Figure 3a; blue trace). These results suggest that AIC enables transport of water that swells the conducting polymer and facilitates volumetric capacitance in a manner that is independent of AIC particle concentration.

Given the ability of AIC to hydrate the underlying conducting polymer layers, we asked what critical particle concentration, for a given particle size, is required to achieve a comparable hydration to an isotropic ion conductor. To answer this question, we developed an image processing protocol to evaluate the particle area of occupancy (density) within the film and its resultant impedance. We prepared films with 5–60% v/w NaPSS particles and PU ion barriers. Each film was imaged at 4 random locations with 4 mm^2^ regions of interest (ROIs). These ROIs were then adaptively binarized to create black and white images where white pixels represent particles and black pixels indicate PU (Figure 3b; inset, Figure S6, Supporting Information). The film thickness in all cases was kept constant and approximately the same as the particle size (10 µm). We then defined the particle density as the ratio of pixels occupied by particles to the total number of pixels (Figure 3b). Based on this analysis, AIC films with larger than 30% particle density, corresponding to 10% v/w particle concentration, can establish a similar effective hydration path as isotropic electrolytes (Figure 3c).

**Figure 4:**
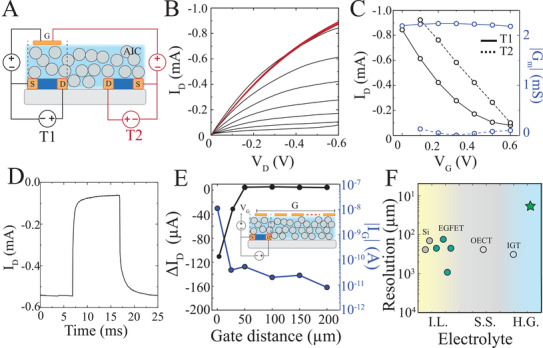
AIC enables high‐density, independently addressable, low crosstalk transistor circuits. A) Cross‐sectional schematic of two adjacent OECTs with AIC as electrolyte. Black wiring highlights transistor (T1) equipped with an independent gate electrode aligned to the surface of the channel while red wiring illustrates the adjacent transistor (T2) connected to T1's gate (which is misaligned to T2's channel). B) Output characteristics of T1 (black) and T2 (red) OECTs (*L* = 250 µm, *W* = 1 mm) with an NaPSS/PU AIC ion membrane (*H* = 10 µm, 60% w/v concentration). Modulation of the transistor output is maintained when the gate electrode is aligned over the channel (black), while dedoping is prevented when the gate and channel are laterally misaligned (red). C) Transfer curves for *V*_D_ = −0.6 V (black) and the corresponding transconductance curves (blue) for aligned gate (solid) and misaligned gate (dashed) electrode. D) Time response of a PEDOT:PSS‐based OECT with NaPSS/PU AIC as electrolyte (60% w/v concentration (*H* = 10 µm, 60% w/v concentration). E) Transistor responses measured as a function of gate electrode lateral distance from the channel for a depletion‐mode transistor. Zero denotes an aligned gate electrode to the channel. The transistor response ΔI_D_ is defined as the difference in drain current when *V*_G_ = 0 V and *V*_G_ = 0.6 V. Inset: schematic illustration of the OECT with multiple gates at various lateral distances away from the channel. Channel is only modulated when gate and the channel are aligned due to the NaPSS/PU AIC (*H* = 10 µm, 60% w/v concentration). F) Comparison of the spatial resolution of solution‐gated transistors in the literature as a function of their electrolyte. Green star denotes AIC (I.L. = ionic liquid and gel, S.S. = solid state, H.G. = hydrogel‐based).

We then integrated AIC with thin film technologies and flexible electronics to evaluate its capacity as an anisotropic electrolyte. We microfabricated arrays of PEDOT:PSS‐based enhancement (normally OFF) and depletion (normally ON) mode OECTs with channel lengths ranging 10^1^–10^3^ µm (Experimental Section). When operated in phosphate‐buffered saline (PBS), these transistors demonstrated typical high‐transconductance, low‐voltage characteristics expected from such electrochemical devices (Figures S7–S10).^[^ ^23^, ^30^, ^31^ ^]^ We then deposited AIC over these characterized transistors. They exhibited similar performance characteristics when operated through AIC compared to PBS, in keeping with the high ion conduction of particles across the AIC film (Figure S7a,b, Supporting Information). However, it is not possible to address individual transistors that share an isotropic electrolyte (PBS) because application of gate voltage (*V*_G_) to any gate will result in drain current (*I*_D_) modulation in all transistors (**Figure** **4**a). This unwanted leakage between transistors impedes formation of circuits with OECTs (Figure S8, Supporting Information). In contrast, the transistors coated with AIC maintained lateral isolation, permitting modulation at any individual transistor's gate without inducing a response at the gates of neighboring transistors (Figure 4b,c, Figure S9, Supporting Information). Notably, this was done without impeding the temporal response of the transistors (Figure 4d). Next, we sought to establish the circuit density that AIC can establish with minimum cross‐talk between adjacent transistors. We simultaneously acquired (*I*_D_) drain and gate currents (*I*_G_) of a single OECT with gates at different distances from the transistor channel (Figure 4e; inset). We showed that a 60% v/w, 10 µm‐based AIC was able to reduce gate current by approximately 2 orders of magnitude at 25 µm offset (Figure 4e). It is possible to achieve electrochemical gating using AIC and an in‐plane gate electrode. This could be accomplished by controlling the size and particle density of conducting particles. Particles or chains of particles equal or larger than channel‐gate distance can be used to establish a lateral ionic path between gate and channel. In turn, this length should be shorter than inter‐transistor spacing to eliminate any conduction across transistors (Figure S10, Supporting Information). Overall, the density of transistors achieved by AIC surpasses the majority of ion‐driven transistors by several orders of magnitude (Figure 4f, Table S3, Supporting Information) and suggests potential to increase the capacity of these transistors in integrated circuits and biosensors.^[^ ^32^, ^33^, ^34^ ^]^

**Figure 5:**
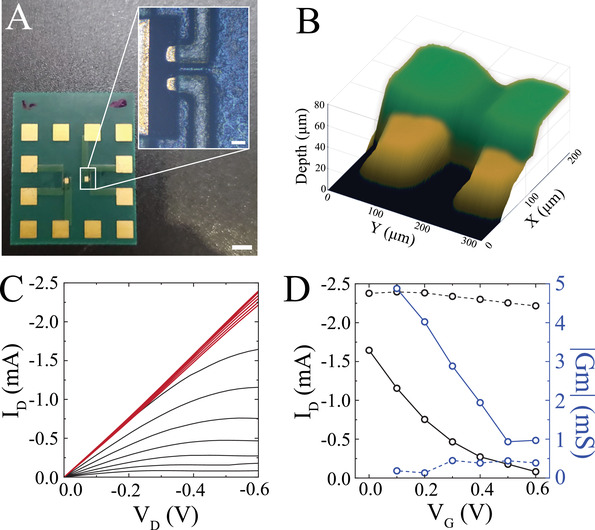
AIC is applicable in flexible electronics with high roughness surfaces. A) Micrograph of an OECT on a flexible PCB substrate with a patterned PEDOT:PSS (*L* = 50 µm, *W* = 100 µm) channel and approximately 50 µm × 50 µm gold contact areas. Scale bar 3 mm. Inset scale bar 50 µm. B) 3D profilometry showing the roughness of flexible electronics substrates. C) Output characteristics of an OECT operating in depletion mode with a NaPSS/PU AIC ion membrane (*H* = 10 µm, 40% w/v concentration). Modulation of the transistor output is maintained when the gate electrode is aligned directly over the channel (black), while gating is prevented when the gate is misaligned (red) 3 mm away from the channel. V_G_ varied from 0 to 0.6 V, in increments of 0.1 V (top to bottom). D) Characteristics of an AIC‐based OECT. Transfer curves for *V*_D_ = −0.6 V (black) and the corresponding transconductance curves (blue) for aligned gate (solid) and misaligned gate (dashed) electrodes.

The ability to merge thin film transistors with existing high throughput printed circuit boards (PCB) and printed electronics is particularly important for development of biosensors, large scale electronics, wearable devices, and bioelectronics because transistors could be directly integrated into the boards while still benefiting from large scale, high yield printing technologies. However, coupling thin film transistors with PCBs is highly challenging because: i) there is no currently available ion conductor for PCBs, and ii) the large surface roughness of PCBs prohibits deposition of continuous thin films. The most widely used PCBs consist of an insulating core layer often made out of flame retardant fibers (FR4) or polyimide (Pi) that are metalized with 12–18 µm thick Cu. The Cu is patterned using an etch process and the FR4/Cu can be stacked to form multilayer boards. A thin layer of anti‐corrosive such as nickel, Au, Sn, or a combination of these materials is used to coat the outer Cu pads. An additional insulating layer of resin, often referred to as a solder–stop layer (10–20 µm), is added for encapsulating interconnects. As a result, such multi‐stack boards will often have 20–100 µm walls that prevent adequate contact between the PCB and a continuous thin film. To investigate the performance of AIC in combination with commercially available PCBs, we first fabricated printed boards with two transistors (50 × 50 µm^2^ channel area; **Figure** **5**a,b). The channels (PEDOT:PSS) of the transistors were then deposited using spin coating. These transistors had similar performance to those that were microfabricated on thin films, as determined by their output characteristics and transconductances in PBS using a PEDOT:PSS‐based gate (Figure 5c,d, black curves). However, in PBS they were not individually addressable as one transistor's gate effectively modulated the drain current of the other (Figure S11, Supporting Information). We synthesized AIC with 10 µm thickness, PSS‐based particles, and PU‐based insulating material. This combination of material and particle size allowed formation of a continuous film that could be applied using a blade coating process. Substituting PBS with AIC, we found that the performance of the transistor remained unchanged, but the adjacent transistors were individually addressable through their contact with the PCB (Figure 5c,d).

**Figure 6:**
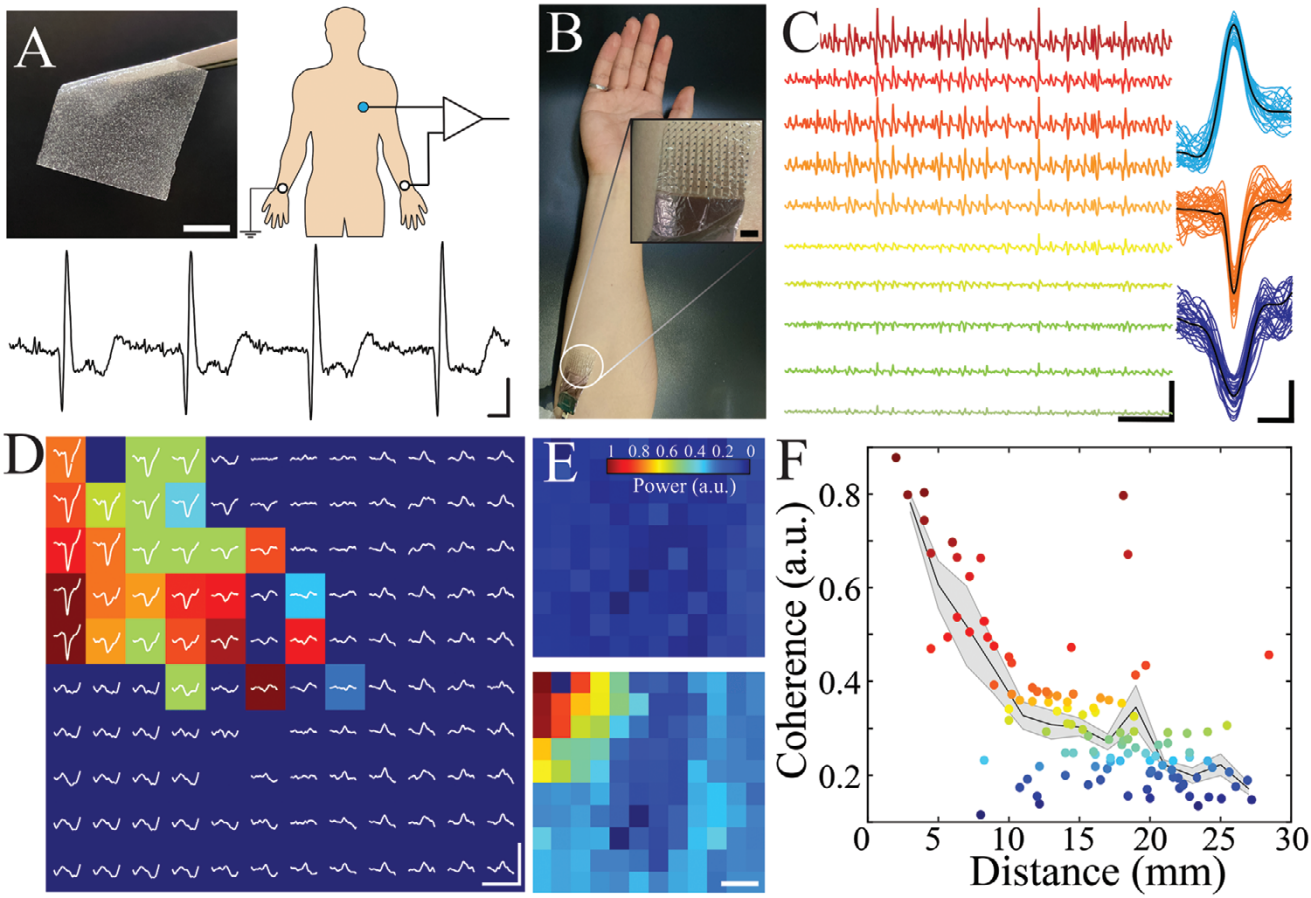
AIC establishes electrically and mechanically stable anisotropic interfaces that enable high spatiotemporal resolution signal transduction in non‐invasive electrophysiology. A) Stretchable and conformable standalone PAAm/Sorbitol/PDMS film (left; 15% w/v, 10% w/v, particle size = 100 µm; scale bar 10 mm). Schematic of EKG measurement with AIC (blue) placed on the surface of the skin over the subject's heart and conventional gel electrodes (white) are placed on the wrists for reference and ground. Scale bar 100 ms, 1 mV. B) The wearable AIC film conforms to skin as an interface that enables high‐resolution surface EMG recordings. High‐density surface EMG is recorded from the flexor digitorum superficialis using a 120‐electrode probe with reference and ground placed over the olecranon. Scale bar 4 mm. C) Sample EMG traces during isometric flexion of the ring finger after filtering (40–900 Hz) and median removal (left). Scale bar 25 ms, 5 mV. Examples of distinguishable waveforms acquired from high‐density EMG recording using the 120‐electrode probe with AIC interface (right). The waveforms are time‐aligned (colored traces) and the averages (black traces) are calculated. Warmer colors represent higher amplitude in arbitrary units. Scale bar 1 ms, 1 mV. D) EMG traces (band‐pass filtered and median removed) from electrodes across the 120‐electrode probe reveal localized distribution of muscle action potential; Scale bar 1 mm, 2.5 mV. Corresponding colormap of latency of the maximal negativity of the spike waveform; * denotes reference electrode for latency measurement. Electrodes that do not exhibit a negative peak are show in dark blue. E) EMG power during relaxation (left) and isometric flexion (right) of the ring finger is measured as the root‐mean‐square (RMS) of the bandpass‐filtered (40–900 Hz) and median‐removed EMG signal for each signal from the electrode array. Warmer colors represent higher relative power in arbitrary units (a.u.). Scale bar 2 mm. F) Coherence of surface EMG activity patterns at millimeter scales during isometric muscle contraction at a frequency band of 40–900 Hz in a healthy subject using a 120‐electrode probe with AIC interface. Coherence values of pairs of electrodes are averaged in bins of 2 mm and plotted as mean ± standard error. Warmer colors represent higher coherence in a.u.

Given the ability of AIC to interface with rough substrates and maintain hydration in the absence of external electrolyte, we next investigated AIC for “dry” biopotential sensing. Wet electrodes are commonly employed because they have a low electrode‐skin interface impedance and offer stable recording.^[^ ^35^ ^]^ However, these wet electrodes can be limited for long‐term recording due to dehydration and skin irritation. Dry electrodes typically overcome these challenges, but at the expense of lower signal‐to‐noise ratio and decreased mechanical stability. AIC is made of biocompatible materials and can be easily modified to gain adhesive properties by addition of sugar alcohols such as D‐sorbitol.^[^ ^35^ ^]^ Therefore, we directly tested AIC's (100 µm PAAm particles in PDMS, with overall 100 µm film thickness; Figure S12, Supporting Information) ability to sense electrocardiography (EKG) and EMG signals from human skin. We found that AIC established a comfortable, mechanically stable interface with skin of the human arm compared to conventional dry electrodes even when the underlying muscle was contracted. No skin preparation other than swabbing with isopropyl alcohol (IPA) was necessary. AIC‐based electrodes acquired single channel EKG and EMG signals with high signal‐to‐noise ratio, establishing the feasibility of this approach for biopotential sensing (**Figure** **6**a–c).

To minimize electrode‐skin impedance, large surface electrodes are often required, limiting the spatial resolution of signals that can be acquired. We investigated whether AIC could enable high spatiotemporal resolution, non‐invasive sensing by affixing a 120 channel, 2 cm × 2.5 cm conformable electrode array to human skin overlying the flexor digitorum superficialis muscle using AIC as an interface material (Figure 6b, Figure S12, Supporting Information). With this approach, multiple differentiable waveforms consistent with motor unit action potentials (MAPs) were identified (Figure 6c). These putative MAPs were strongly elicited by isometric contraction of the underlying muscle, and waveforms displayed physiologically consistent latency and spatial distribution across the electrode array (Figure 6d–e). These results indicate that AIC permits independent sensing from multiple closely spaced (2 mm) electrodes. Indeed, we found that coherence of high frequency (40–900 Hz) signals acquired by the AIC‐based array dropped rapidly with distance from the index electrode (Figure 6f), suggesting that increased density of spatial sampling could yield additional biologically relevant data.

## 3 Discussion

We developed an anisotropic ion conductor using a particulate composite material structure. Ion conducting particles allow effective ion transport across the film, and the insulating scaffold prevents lateral conduction. Particle size and density were tuned to determine the effective spatial resolution for anisotropy. When deposited over the channel of a transistor, AIC enabled transconductance and rise times comparable to use of a conventional electrolyte. Furthermore, application of AIC did not prevent swelling of conducting polymers, preserving their volumetric capacitance. AIC‐based films had stable long‐term anisotropy and allowed patterning‐free independent gating of transistors at a density equivalent to channel length.

AIC can be created using combinations of various hydrogels and biocompatible resins, allowing for further tuning of properties for different applications. The synthesis process is scalable and environmentally friendly. Deposition of AIC can be performed using conventional solution processing approaches, such as spin and blade coating. It is also potentially amenable to large‐scale printing technologies. Therefore, AIC constitutes an optimal solution for establishing organic integrated circuits.

We investigated the use of AIC for non‐invasive biopotential sensing from the human skin and found that it formed a mechanically stable, dry, and comfortable interface. No adhesives were required for application, and high‐quality electrophysiologic data was obtained. AIC‐based interface devices acquired high spatiotemporal resolution EMG signals from densely packed independent sensors (2 mm pitch), and the prevention of cross‐talk was demonstrated by detection of individual MAPs with unique topography across the array. AIC could therefore benefit monitoring approaches that require long‐term electronic contact with tissue.^[^ ^36^, ^37^ ^]^ Future work is necessary to characterize the effect of mechanical strain on anisotropy. Such investigation is particularly important for applications involving elastic scaffolding material and stretchable circuits.

In addition to signal transduction, AIC could facilitate development of neuromorphic devices based on organic materials because it permits the dense integration of transistors necessary for creation of multiple synaptic connections.^[^ ^38^, ^39^ ^]^ The properties of AIC position it to enable implementation of a wide range of organic bioelectronics and enhance their translation to human health applications.

## 4 Experimental Section

### Materials

PEDOT:PSS (Clevios PH1000) was acquired from Heraeus. Ethylene glycol, chitosan (50–190 kD, 75–85% deacetylated), (3‐Glycidyloxypropyl)trimethoxysilane (GOPS), 4‐dodecyl benzene sulfonic acid (DBSA), D‐sorbitol (BioUltra ≥99.5%), 1 wt% polyethylenimine (PEI) diluted in deionized water (1:10), glycerol, PBS, sodium poly(styrene sulfonate), gelatin (from bovine skin), polyacrylamide/acrylate hydrogel (2.5 mm granule) and epoxy embedding medium kit were purchased from Sigma Aldrich. Rigid casting polyurethane (Fabri‐Cast 50) was bought from Specialty Resin & Chemicals. Polydimethylsiloxane (SYLGARD 184) was purchased from Dow.

### Particle Preparation

Ion conducting particles were prepared by grinding the material into finer sizes in a ball mill. The chosen particle material was poured into a 50 mL centrifuge tube. Stainless steel beads with diameters ranging from 3.17–6.35 mm were placed into the centrifuge tube. Enough isopropyl alcohol was added to submerge the particles and steel balls. The centrifuge tube was capped and sealed with Parafilm to prevent leaks, and the tube was mounted onto an automatic shaker for ball milling. After grinding the particles, the balls were removed from the mixture. Excess isopropyl alcohol was removed from the mixture via evaporation until the particles were completely dried. The dry powder was filtered through a set of sieves with varying mesh size from 60 to 100 µm to obtain the particle size distributions of interest. Smaller particles (1–10 µm diameter) were obtained by milling for prolonged periods of time. However, dry sieving became ineffective for small sizes as the mesh was easily clogged. Likewise, wet sieving was not used because the unstable alcohol‐based dispersion caused aggregation, preventing clumped particles from passing through the sieve.

### Polymer/Particle Blend Preparation

Blends of particulate composites were prepared by mixing the particles of the desired size range into the resin of the scaffold polymer. For PDMS, the PDMS elastomer was mixed with the PDMS hardener at a 10:1 mass ratio. Prior to adding particles, the PDMS resin was degassed in a vacuum chamber until no bubbles were visible. For polyurethane, part A (resin) and B (hardener) components were mixed in a 1:1 ratio, and the mixture was deposited within the 2 min working time, after which the polyurethane began to cure. For epoxy, equal portions of the epoxy embedding medium, hardener MNA, hardener DDSA, and accelerator DMP were mixed. Particles were added by mass to a volume of polymer resin. The particulate composite concentration was calculated as the percentage of the weight/volume ratio.

### Film Deposition

The particle and scaffolding resin mixture were deposited onto a substrate via blade coating. A blade coater with a known gap size was used to set the thickness of the AIC film. After coating, the bladed solution was put under a vacuum at 90 kPa to remove air bubbles and was then baked at 120 °C to accelerate the curing process. Bake times varied depending on the type of scaffolding material: 1.5 h for PDMS, 2 min for polyurethane, and 30 min for epoxy.

### Pixel Distribution of Particulate Films

AIC films with NaPSS particles (roughly 10 µm diameter) in polyurethane were deposited onto glass slides with a 10 µm gap blade coater for 10% w/v and 60% w/v concentrations. The films were imaged at 50× magnification in grayscale. Each sample image was analyzed in *n* = 3 random regions of the image for their pixel intensity distributions. The pixel intensity values of the cropped images were binned into 30 bin histograms and fitted into Gaussian distributions. All images were processed using the MATLAB Image Processing Toolbox.

### Particle Size

Clean glass slides were lightly dusted with particles by sieving previously size‐filtered particles. Each glass slide was imaged at 50× magnification at multiple sections and converted to grayscale such that particles were assigned darker shades. Images were then converted to binary using locally adaptive thresholding that discarded pixel intensity values depending on a sensitivity parameter. Transparent regions of particles that created holes due to reflection in the binarized image were filled. The edges of the particles, the area, equivalent diameter, and major and minor axes of a particle were defined from a single connected region. Detected particles with substantially lower size than the sieve mesh size (e.g., 60–75 µm) were considered as noise and excluded from the data set. Similarly, connected regions with equivalent diameters substantially larger than expected were considered as particles in contact with one another, and excluded from the data. The particle size distribution was binned into a histogram and fitted into a normalized Gaussian distribution. All images were processed using the MATLAB Image Processing Toolbox.

### Electrochemical Impedance

Electrochemical impedance spectroscopy (EIS) was performed with a Gamry Reference 600+. Electrochemical impedance was measured in potentiostatic mode with 100 mV root‐mean‐square (RMS) and frequency range from 1 Hz–5 MHz (10 points per decade). AIC samples were deposited on Au electrodes patterned on plastic or glass substrates, spaced 0.25, 0.5, 1, and 2 mm apart. PEDOT:PSS was patterned (via spin‐coating at 2000 RPM) over 2 mm × 5 mm Au electrode areas to provide a mixed electron and ion‐conducting electrode that minimizes impedance contributions by electric double‐layer capacitance. EIS was measured using a two‐electrode configuration and a 1× PBS solution was used to hydrate AIC samples. For through‐plane impedance measurements, excess PBS was placed over the AIC films such that there was a conductive pathway for a gold electrode placed over the film. The gold electrode was used as the working electrode, while PEDOT:PSS coated electrodes served as the counter electrodes in vertical impedance measurements. For in‐plane impedance measurements, excess solution was removed from the surface of the AIC to prevent shorting, and lateral PEDOT:PSS‐based electrodes on the substrate were used as the working and counter electrodes.

### Hydration Stability Measurement

Daily EIS measurements were performed over a 30 day period to show the hydration and electrical stability of encapsulated AIC samples. PAAm/PU (H = 20 µm, 10% w/v concentration) were deposited on a glass substrate with PEDOT:PSS‐coated gold electrodes. The AIC was wet with 1× PBS adequately for high vertical ionic conductivity, but not excessively wet to avoid a lateral ion conduction pathway. Then, another glass plate with patterned Au electrodes was laminated on top of the AIC film such that the electrodes on the top and bottom were aligned. Finally, polyurethane was used to seal the edges and glue the top plate onto the AIC. This created a water‐tight seal to entrap moisture within the PAAm particles. EIS was conducted between the bottom lateral PEDOT:PSS‐coated electrodes (*L* = 250 µm) for the in‐plane impedance, while the through‐plane impedance was measured between the top and bottom electrodes.

### Transistor Characterization

Current–voltage characteristics were obtained using two channels of a Keysight B2902A Precision Source/Measurement Unit (SMU). The first channel (*V*_D_) applied a linear sweep from 0 V to −0.6 V, (−0.03 V step). The second channel (*V*_G_) applied a constant voltage from 0 to 0.6 V, incrementing in steps of 0.1 V. Electrical characteristics were measured using Ag/AgCl gate electrodes for horizontal OECTs and using a patterned gold electrode for vertical IGTs. Temporal responses of OECTs were measured by a Keysight B2902A that applied a constant *V*_D_ (−0.6 V) and produced a pulse train (*V*_G_ = 0.6 V) for measurements. Temporal responses measurements of IGTs used a Keysight B2902A to supply constant *V*_D_ (−0.6 V), a Keysight 33500B function generator to pulse a square wave (*V*_high_ = 0.6 V, *V*_low_ = 0 V) at various frequencies on the gate electrode, and a Keysight DSOX2002A oscilloscope to measure current passing through a 100 Ω resistor at the drain of the transistor.

### OECT Fabrication

Depletion‐mode OECTs were fabricated by patterning PEDOT:PSS channels onto Au source and drain electrodes. PEDOT:PSS was spin‐coated at 2000 RPM onto a glass substrate with Au electrodes and a polyimide mask. Peel‐off of the polyimide mask resulted in a PEDOT:PSS channel (*L* = 250 µm, *W* = 1 mm) and 2 mm × 1 mm contact areas. AIC‐based OECTs were prepared by blade coating NaPSS/PU (*H* = 10 µm) over the PEDOT:PSS channels, and hydrated with 1× PBS.

### IGT Fabrication

A 1.5 µm‐thick parylene C layer was coated on double side polished quartz wafers (100 mm outer diameter, thickness of 1 mm) using an SCS Labcoater 2. Metal electrodes and interconnects were patterned through a metal lift‐off process. A 10 nm thick Ti adhesion layer, followed by a 150 nm thick Au layer were deposited (Angstrom EvoVac Multi‐Process). A second layer of parylene C (insulation layer), followed by an additional sacrificial layer of parylene C (for the subsequent peel‐off process) were deposited similarly to the first layer. During chemical vapor deposition of the second Pa‐C layer, 3‐(trimethoxysilyl)propyl methacrylate (A‐174 silane) was used to enhance adhesion between the first and the second layer. Spin‐coated anti‐adhesion agent (5 wt% Micro‐90 diluted in deionized water) reduced the adhesion between the second and third layers. The stacked layers were patterned with a 4.6 µm‐thick AZ9260 photoresist and dry etched with an O_2_ plasma reactive ion etching process (Oxford Plasmalab 80) to shape the IC electrodes and electrical contact pads. The PEDOT:PSS formulation was prepared based on previously described methods.^[^ ^9^, ^20^ ^]^ Spin‐coated PEDOT:PSS films were patterned by peeling off the third parylene layer. For enhancement mode transistors, a blend of PEDOT:PSS/PEI blend was used as the channel material, similar to previously described methods^[^ ^9^ ^]^

### Standalone AIC synthesis

Stretchable and conformable AIC films were designed by using PDMS elastomer as the insulating scaffold. PAAm particles were chosen because the hydrogel was water insoluble, stays embedded into the PDMS scaffold, and swells when hydrated to form good contact. PAAm particles filtered between 75 and 100 µm sieves were used and a 15% w/v particle‐resin blend was blade coated with a 100 µm gap. The blend also consisted of 10% w/v sorbitol particles of the same size as an adhesion‐promoting sugar alcohol. Polystyrene was used as the substrate for its low surface energy. The composite was cured at 70 °C for 3 h rather than the typical 120 °C of PDMS to avoid thermal deformation of polystyrene and the melting point of D‐sorbitol (sugar alcohol used as biocompatible adhesive). After solidification, composites were peeled off the polystyrene substrate to obtain a standalone AIC film, used in signal transduction applications.

### EMG Recordings

All experiments were performed in compliance with the Institutional Review Board at Columbia University. Informed consent was obtained from all participants. High‐density surface electromyographic (hd‐sEMG) recordings were performed on healthy volunteers (*n* = 3, all right‐handed). The Pa‐C substrate probe (10 × 12 channels, 2 mm spacing, Au electrode size: 500 µm × 500 µm) was placed on the right arm, targeting the flexor digitorum superficialis, with conventional gel electrodes for reference and ground, targeting the olecranon area of the right arm and left arm respectively. Before each recording, the skin at the recording site was cleaned using 50% v/v IPA. Standalone AIC (2 cm × 2.5 cm) was placed on the cleaned skin and sprayed with 1× PBS for better contact, and the layer was allowed to dry at room temperature for 30 s before the probe was placed on the skin. Signals were acquired by sampling at 20 kHz, using a custom board incorporating an RHD2164 die (Intan Technologies). Signals were visualized in real‐time with the RHD2000 Interface Software. Data were stored for analysis in a 16‐bit format and analyzed with MATLAB (MathWorks).

The subjects were asked to perform an isometric flexion of the ring, middle, and index fingers. During the task, the subjects were asked to relax their right hand on the desktop and apply constant force (8 N) to press the force meter (NEXTECH DFS 50N) fixed right above the fingertip.

### Motor Unit Analysis

Band‐pass filtering (40–900 Hz) and median removal were applied to the recorded HD‐sEMG. Spikes with similar waveforms were collected and time‐aligned to the extrema from each channel. Average waveforms were calculated within each group. The RMS of band‐pass filtered and median‐removed EMG were calculated for each channel during a resting and isometric flexion session respectively. Spike localization and traveling were quantified by the latency of the maximal negativity of the spike waveform.^[^ ^40^ ^]^ The time latency was calculated by subtracting the maximal negativity time point on each channel from the maximal negativity time point on the reference channel. Coherence was calculated using multitaper time‐frequency analysis (Chronux; http://chronux.org/).

## Conflict of Interest

The authors declare no conflict of interest.

## Author contributions

D.K., J.N.G., and R.Y. conceived the project. RY synthesized materials, fabricated devices, and performed electrical measurements. R.Y., H.Y., and J.N.G. designed and performed electrophysiological recordings and analysis. C.C. and Z.Z. contributed to device fabrication and electrical, electrochemical, and topography measurements, and O.R. performed electron microscopy. All authors contributed to writing the paper.

## Supporting information

Supporting Information

## Data Availability

The data that support the findings of this study are available from the corresponding author upon reasonable request.
