# Supplementary material for: Anisotropic Ion Conducting Particulate Composites for Bioelectronics
Source: Adv Sci (Weinh). 2022 Jan 27;9(9):2104404. doi: 10.1002/advs.202104404 (PMC8948554; doi:10.1002/advs.202104404)
Supplement: Supplementary file 1 — Supporting Information [file ADVS-9-2104404-s001.pdf]

ADVANCED SCIENCE
Open Access

Supporting Information

for *Adv. Sci.*, DOI 10.1002/advs.202104404

Anisotropic Ion Conducting Particulate Composites for Bioelectronics

*Dickson R. Yao, Han Yu, Onni J. Rauhala, Claudia Cea, Zifang Zhao, Jennifer N. Gelinas and Dion Khodagholy**

WILEY-VCH

ADVANCED SCIENCE
Open Access

Supporting Information

for *Adv. Sci.*, DOI: 10.1002/advs.202104404

Anisotropic ion conducting particulate composites for bioelectronics

*Dickson R. Yao, Han Yu, Onni J. Rauhala, Claudia Cea, Zifang Zhao, Jennifer N. Gelinas, and Dion Khodagholy**

Anisotropic ion conducting particulate composites for bioelectronics

Dickson R. Yao[1], Han Yu[1], Onni J. Rauhala[1], Claudia Cea[1], Zifang Zhao[1], Jennifer N. Gelinas[2,3], Dion Khodagholy*[1]

1. Department of Electrical Engineering, Columbia University, New York, NY 10027, USA
2. Department of Neurology, Columbia University Medical Center, New York, NY 10032, USA.
3. Institute for Genomic Medicine, Columbia University Medical Center, New York, NY 10032, USA

*Corresponding authors:
Dion Khodagholy: dk2955@columbia.edu

Keyword:
Organic bioelectronics, anisotropic electrolyte, integrated organic electrochemical transistors

Abstract:
Acquisition, processing and manipulation of biological signals requires transistor circuits capable of ion to electron conversion. However, use of this class of transistors in integrated sensors or circuits is limited due to difficulty in patterning biocompatible electrolytes for independent operation of transistors. We hypothesized that it would be possible to eliminate the need for electrolyte patterning by enabling directional ion conduction as a property of the material serving as electrolyte. Here we develop the anisotropic ion conductor (AIC) as a soft, biocompatible composite material comprised of ion-conducting particles and an insulating polymer. AIC displays strongly anisotropic ion conduction with vertical conduction comparable to isotropic electrolytes over extended time periods. AIC allows effective hydration of conducting polymers to establish volumetric capacitance, which is critical for the operation of electrochemical transistors. AIC allows dense patterning of transistors with minimal leakage using simple solution-based deposition techniques. Lastly, AIC can be utilized as a dry, anisotropic interface with human skin that is capable of non-invasive acquisition of individual motor action potentials. The properties of AIC position it to enable implementation of a wide range of large-scale organic bioelectronics and enhance their translation to human health applications.

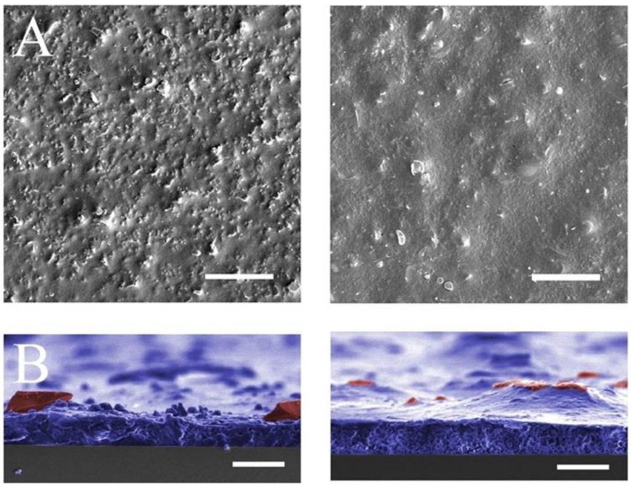

Supplementary Figure 1: SEM images of NaPSS/PU composites deposited with a 10 µm blade-coater gap. False colored images highlight NaPSS particles (red) and PU scaffold (blue) on a glass substrate (gray). Ion conducting particles embedded in an insulating scaffold form ionically conductive pathways through the plane of the material.

A) Top-down SEM images of NaPSS/PU composites (H = 10 µm) at two concentrations. Left: 40% w/v; right: 60% w/v. Scale bar, 50 µm, 50 µm.

B) Cross-sectional SEM images of NaPSS/PU composites (H = 10 µm) at two concentrations. Left: 40% w/v; right: 60% w/v. Scale bar 10 µm, 10 µm.

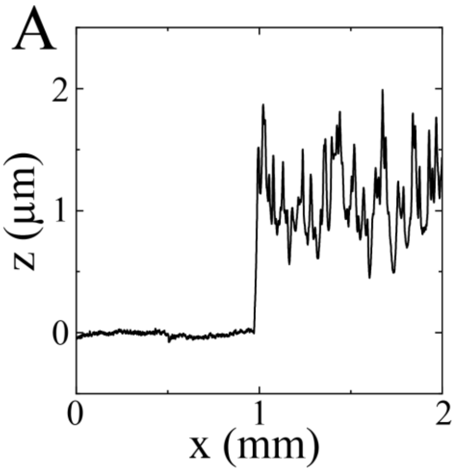

Supplementary Figure 2. Two-dimensional mechanical profilometry measurements on NaPSS/PU (H = 1 µm) films showing ± 0.25 µm surface roughness.

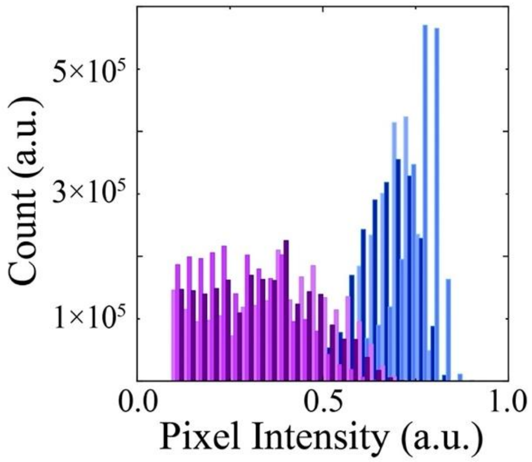

Supplementary Figure 3: Histogram of grayscale pixel intensity for AIC films presented in **Figure 1D**.

Histograms (# bins = 32) of the pixel intensity for micrographs of NaPSS/PU composites (H = 10 µm) at 10% w/v (blue) and 60% w/v (purple) concentrations. Overlapping histograms of pixel intensity at n = 3 different regions for each particle concentration indicate that particles were evenly distributed throughout the film. Pixel intensity distributions in Figure 1F were generated from fitting these histograms to Gaussian distributions.

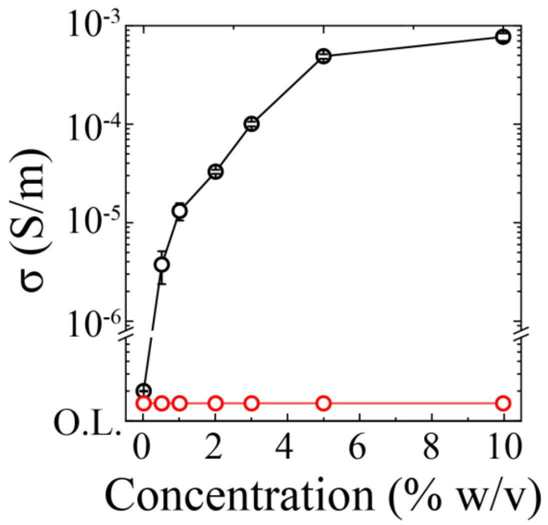

Supplementary Figure 4: The vertical ionic conductivity of AIC is tunable as particle concentration is varied.

The ionic conductivities of NaPSS/PDMS AIC (H=100 μm) reported here correspond to **Figure 2A**. Values for conductivity were calculated based on the dimensions of 2 mm × 5 mm electrodes used for measurements. Vertical ionic conductivity (black) is tunable, while horizontal ionic conductivity (red) is not reported due to the impedance being out-of-range from the detection limit of the measurement instrument. O.L., overload.

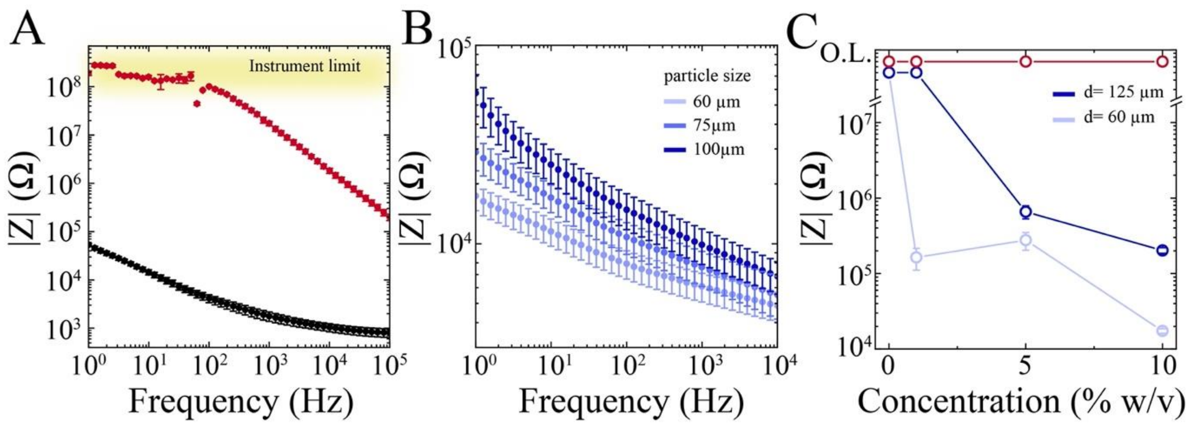

Supplementary Figure 5: Electrochemical impedance spectroscopy of anisotropic ion conducting films.

A) Impedance spectrum of NaPSS/PDMS (10% w/v concentration of 100 µm particles) measured between horizontally spaced electrodes (red, d = 250 µm) and vertical electrodes (black, d = 100 µm). Although the horizontal electrode spacing was only 2.5 times larger than the vertical electrode spacing, the horizontal and vertical impedances differed by several orders of magnitude across the entire frequency range. The horizontal impedance between 1–100 Hz was beyond the detection limit of the measurement.

B) Vertical impedance spectra of the NaPSS/PDMS films (particle size = 60 µm, 75 µm, 100 µm; from light to dark blue) were comparable despite varying the particulate composite parameters. Particle sizes and film thicknesses were kept equal in each case to maintain anisotropy.

C) Vertical (shades of blue) and horizontal (red) impedances as a function of particle concentration for NaPSS/PDMS films (particle size = 60 µm) with different thicknesses.

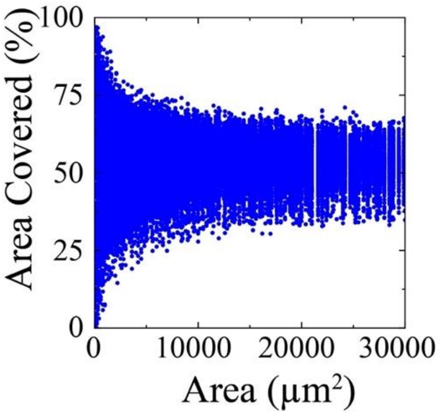

Supplementary Figure 6: Ion conducting particle's exposed surface area determines the vertical impedance of the ion membrane at high frequency.

Simulation of the percentage of area covered by NaPSS/PU (particle size = 10 µm, 60% w/v concentration) AIC particles. Small areas had high variance, whereas larger areas average to approximately 50% area covered.

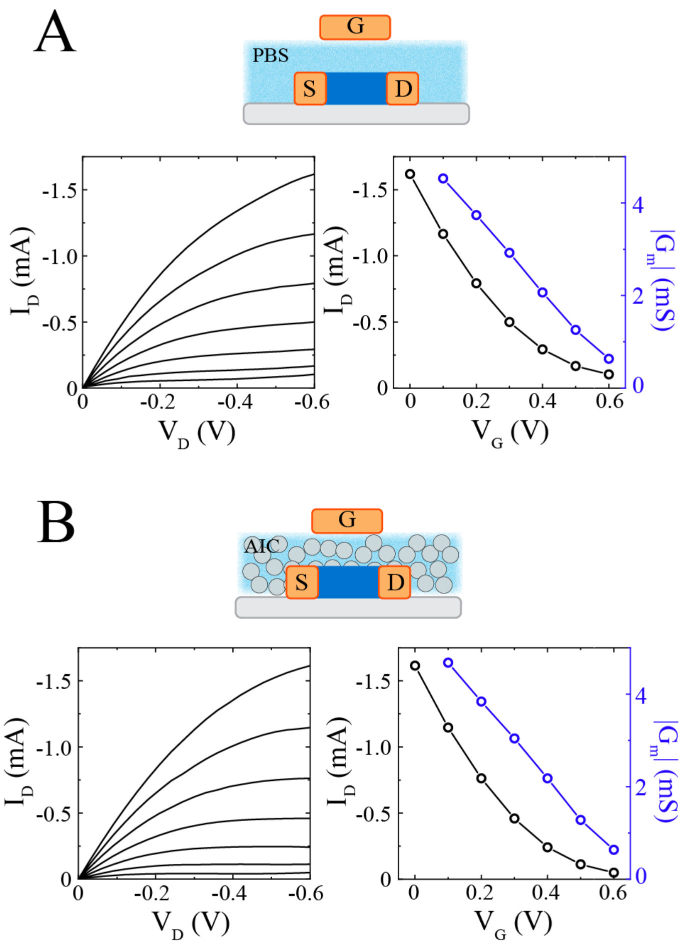

Supplementary Figure 7: AIC ion membranes applied to OECTs does not diminish the performance of the transistor.

A) Left: Output characteristics of an OECT operating in depletion mode (L = 250 µm, W = 1 mm) with a 1× PBS electrolyte (left). V_G varied from 0 to 0.6 V, in increments of 0.1 V (top to bottom). Right: Transfer curves for V_D = −0.6 V (black) and the corresponding transconductance curves (blue).

B) Left: Output characteristics of the same OECT in (A) with an NaPSS/PU AIC ion membrane (H = 10 µm, 60% w/v concentration) replacing the liquid electrolyte (left). V_G varies from 0 to 0.6 V, in increments of 0.1 V (top to bottom). Right: Transfer curves for V_D = −0.6 V (black) and the corresponding transconductance curves (blue).

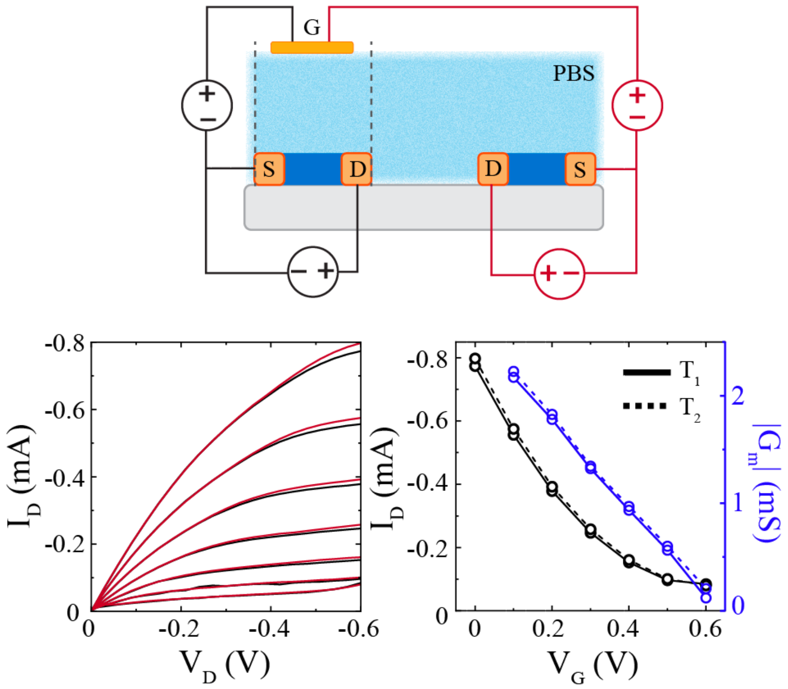

Supplementary Figure 8: Shared liquid electrolyte short-circuits adjacent transistors such that gating one transistor switches neighboring transistors.

Left: output characteristics of a depletion mode OECT (L = 250 µm, W = 1 mm) with of a 1× PBS electrolyte. The transistor output was nearly identical when a gate electrode was aligned (at G1) directly over the channel (black) and when the gate was misaligned (red) 2.5 mm away from the channel (at G2).

Right: transfer curves for $V_D = -0.6$ V (black) and the corresponding transconductance curves (blue), for aligned gate (solid) and misaligned gate (dashed) electrodes.

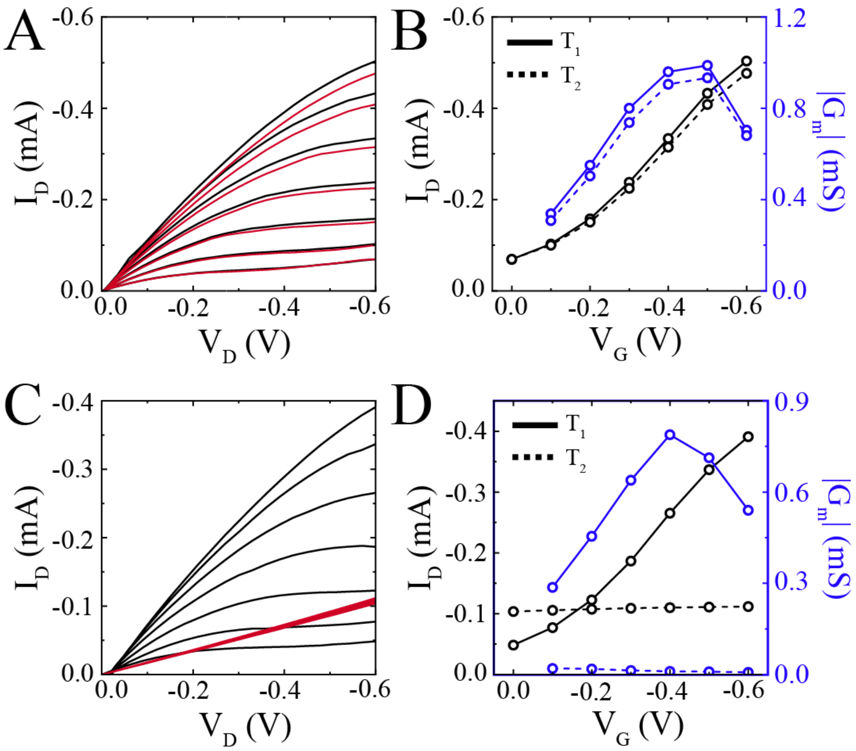

Supplementary Figure 9: AIC is compatible with enhancement-mode OECTs. AIC enables high-density transistor arrays that can incorporate both enhancement-mode and depletion-mode transistors for organic integrated circuits.

A) Output characteristics of an OECT (L = 250 µm, W = 1 mm) operating in enhancement mode (PEDOT:PSS/PEI blend as channel material), with a 1×PBS electrolyte. The transistor output with an aligned gate (black) and gate with 2.5 mm misalignment (red) was nearly identical. V_G varied from 0 to -0.6 V, in increments of -0.1 V (bottom to top).

B) Characteristics of a 1×PBS-based enhancement-mode OECT. Transfer curves (black) for $V_D = -0.6$ V and the corresponding transconductance curves (blue), for aligned gate (solid) and 2.5 mm misaligned gate (dashed) electrode.

C) Output characteristics of an enhancement-mode OECT with NaPSS/PU AIC (H = 10 µm, 60% w/v concentration). The enhancement mode transistor stays normally off when the gate is misaligned (red), while switching the transistor is possible when the gate is aligned (black).

D) Characteristics of an AIC-based OECT operating in enhancement mode. Transfer curves (black) for $V_D = -0.6$ V and the corresponding transconductance curves (blue), for aligned gate (solid) and 2.5 mm misaligned gate (dashed) electrode.

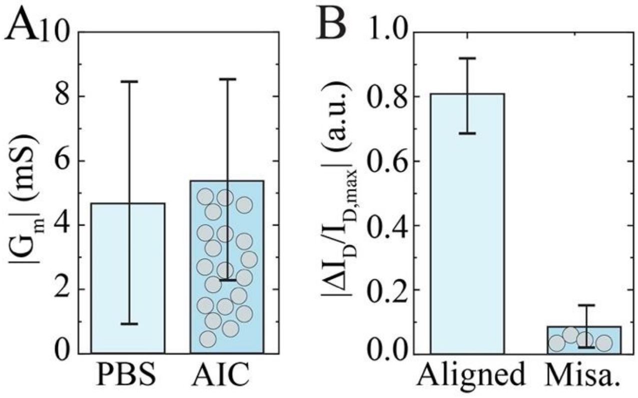

Supplementary Figure 10. AIC enables independent gating in a dense array of high-speed electrochemical transistors without compromising their performance. This is demonstrated on a 4-inch wafer with over 100 transistors comprised of individual lateral PEDOT:PSS-based gate electrodes.

A) Transconductance of IGTs with a liquid 1×PBS electrolyte and an NaPSS/PU (particle size = 10 μm, 60% w/v concentration) ion membrane for 8-12 μm channel lengths. Bars represent mean ± standard deviation.

B) Normalized response of transistors when gated by aligned/misaligned gates for 8-12 μm channel lengths. Bars represent mean ± standard deviation.

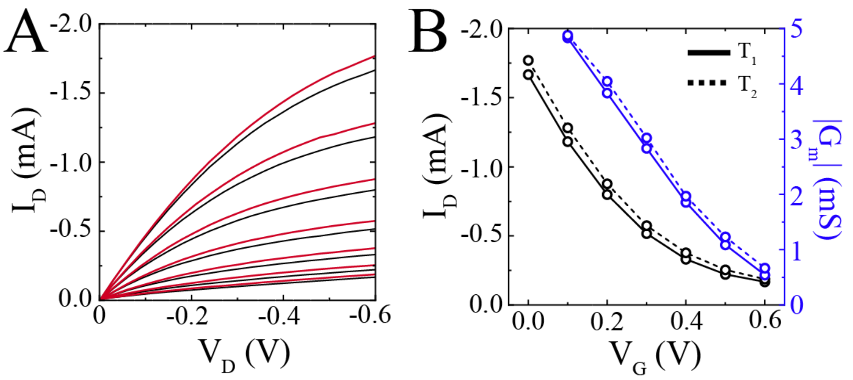

Supplementary Figure 11: Leakage occurs between OECTs patterned on flexible PCB substrates when a shared electrolyte is used.

A) Output characteristics of a 1×PBS-based OECT. Output of the transistor with an aligned gate electrode (black curves) and 3 mm misaligned gate (red curves) was similar. V_G varied from 0 to 0.6 V, in increments of 0.1 V (top to bottom).

B) Characteristics of an OECT patterned on a rough plastic surface, with a 1×PBS liquid electrolyte. Transfer curves (black) for $V_D = -0.6$ V and the corresponding transconductance curves (blue), for aligned gate (solid) and 3 mm misaligned gate (dashed) electrode.

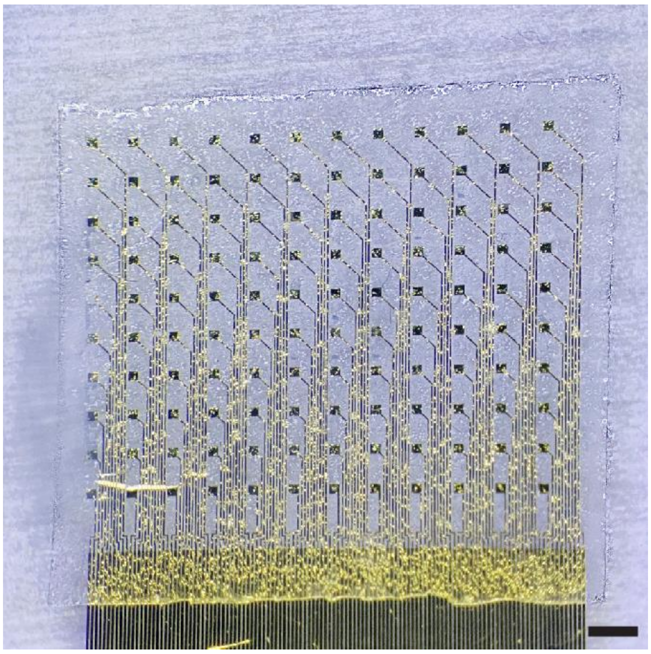

Supplementary Figure 12: Standalone AIC films can be integrated with conformable surface EMG arrays.

PAAm/Sorbitol/PU AIC (15% w/v, 10% w/v, particle size = 100 μm) laminated onto a high-density electrode array for EMG recordings. Scale bar 2 mm.

**Sieve Opening - Lower Bound**	**Sieve Opening - Upper Bound**	**Mean (μ)**	**Standard Deviation (σ)**
N/A	60 µm	37.46 µm	12.98 µm
60 µm	75 µm	75.66 µm	9.31 µm
75 µm	100 µm	103.9 µm	15.84 µm

Supplementary table 1: Mean and standard deviation of the particle size distributions corresponding to Figure 1B.

Particle Concentration	Amplitude Constant	Mean pixel intensity (0-256)	Standard Deviation (0-256)
5%	0.02381	178.30	20.82
5%	0.01983	174.00	28.02
5%	0.03226	196.10	15.31
40%	0.00874	92.88	73.72
40%	0.01209	58.65	61.58
40%	0.00987	72.85	71.82

Supplementary table 2: Gaussian fit parameters and statistics corresponding to the curves in Figure 1D that demonstrate the uniform spread of particles in the scaffold. Zero denotes the darkest pixel value whereas 256 reflects the brightest pixel value for reported means and standard deviations.

	**Transistor**	**Electrolyte**	**Patterning Method**	**Transistor Spacing (μm)**	**Reference**
1	EGOFET (CNT TFT)	Ion gel	Printing	500	[38]
2	EGOFET (Zinc oxide)	Ion gel	Stencil	500	[39]
3	EGOFET (organic)	Ion gel	Printing	1000	[40]
4	SGFET (graphene)	Ionic liquid	Dry etching	400	[41]
5	IGT	Ionic liquid	Lithography	750-1000	[9,10]
6	OECT	*Solid*	Printing	*500*	[15]
7	OECT	*PSS/PU*	--	*25*	This work

Supplementary table 3: Device type, electrolyte, patterning method of electrolyte, transistor spacing for devices presented in Figure 4F.

Acknowledgments

This work was supported by Columbia University School of Engineering and Applied Science as well as Columbia University Medical Center, Department of Neurology and Institute for Genomic Medicine. The device fabrication was performed at Columbia Nano-Initiative. This work was supported by the National institute of health grants (1U01NS108923-01, R01NS118091, R21 EY 32381-01), National Science Foundation CAREER award (1944415), and EAGER (2027135). We thank Youry Borisenkov, J. Vichiconti, N. Ariel-Sternberg (CNI), and all Khodagholy and Gelinas laboratory members for their support.

Author contributions

DK, JNG and RY conceived the project. RY synthesized materials, fabricated devices and perform electrical measurements. RY, HY and JNG designed and performed electrophysiological recordings and analysis. C.C and ZZ contributed to device fabrication and electrical, electrochemical and topography measurements and OR performed electron microscopy. All authors contributed to writing the paper.

Data Availability

All data needed to evaluate the conclusions in the paper are present in the paper and/or the Supplementary Materials. Additional data related to this paper may be requested from the authors.

Conflict of Interest

All authors must declare financial/commercial conflicts of interest. If the authors have no conflicts of interest, this should be stated.
